# Non-traditional lipid parameters are independent predictors of the location, distribution, and stroke events of moderate-to-severe intracranial and extracranial atherosclerotic stenosis

**DOI:** 10.3389/fneur.2025.1564966

**Published:** 2025-07-09

**Authors:** Yin Fei Huang, Zhen Xing Liu, Kuan Cen, Ren Wei Zhang, Qiao Yuan Xiang, Qi Cai, Chun Jiao Yang, Lei Luo, Hai Long Xu, Yu Xie, Yu Min Liu

**Affiliations:** ^1^Department of Neurology, Zhongnan Hospital Affiliated to Wuhan University, Wuhan, Hubei, China; ^2^Department of Neurology, Yiling Hospital of Yichang City, Yichang, Hubei, China

**Keywords:** non-traditional lipid parameters, intracranial atherosclerotic stenosis, extracranial atherosclerotic stenosis, Castelli's risk index-II, stoke

## Abstract

**Objective:**

Moderate-to-severe stenosis has been identified as a significant risk factor for stroke recently. This study aims to investigate the relationship between non-traditional lipid parameters and the location and distribution of stenosis, as well as symptomatic events, in patients with moderate-to-severe intracranial atherosclerotic stenosis (ICAS) and extracranial atherosclerotic stenosis (ECAS).

**Methods:**

This study analyzed correlation between non-traditional lipid parameters and moderate-to-severe ICAS and ECAS concerning stenosis location, distribution, and the presence or absence of symptoms. Logistic models and restricted spline analysis were utilized to explore the relationship between Castelli's risk index-II (CRI-II) and the occurrence of stroke events.

**Results:**

The present study comprised 1,030 participants, of whom 143 were non-stenotic and 887 were patients with moderate-to-severe stenosis. The study focuses on the latter and indicated statistically significant differences in AIP, LCI, RC, AC, CRI-I, and CRI-II among the three groups of ICAS, ECAS, and combined ICAS and ECAS (*P* = 0.012, 0.005, 0.013, 0.009, 0.009, 0.032, respectively). Lipid parameters for ICAS were generally higher than those for ECAS. Remnant cholesterol (RC) exhibited a discrepancy among the anterior, posterior, and combined anterior and posterior circulation stenosis groups (*P* = 0.047). Logistic regression analysis revealed that CRI-II (Odds ratio [OR] = 1.20, Confidence interval [CI] 1.03–1.40, *P* = 0.009) and low-density lipoprotein cholesterol (LDL-c) (OR = 1.21, CI 1.03–1.42, *P* = 0.011) demonstrated remarkable elevations in symptomatic stenosis patients compared to patients without symptoms. After adjusting for potential confounding factors, CRI-II remained an independent risk factor for symptomatic stenosis. Furthermore, multivariate spline regression modeling elucidated that an augmented risk of stroke events in moderate-to-severe stenosis was associated with an elevated CRI-II. As CRI-II elevated, the risk of stroke events increased progressively.

## Background

Stroke is the second leading cause of mortality worldwide and a pivotal risk factor for mortality and disability in adults. Currently, ischemic stroke has been the predominant form of stroke (1, 2). Intracranial atherosclerotic stenosis (ICAS) and extracranial atherosclerotic stenosis (ECAS) have represented a significant cause of ischemic stroke (3, 4). Atherosclerotic stenosis is classified by the degree of stenosis as mild (<49%), moderate (50%−69%), and severe (70%−99% or occlusion) to facilitate clinical diagnosis and treatment (5). Moreover, moderate-to-severe stenosis is a vital risk factor for stroke events, making it crucial to elucidate its etiology.

Prior research has indicated that irregularities in lipid metabolism are associated with the development of atherosclerosis. However, recent studies challenge the notion that traditional lipid parameters, such as total cholesterol (TC), triglycerides (TG), low-density lipoprotein cholesterol (LDL-c), and high-density lipoprotein cholesterol (HDL-c), are the most accurate predictors of cardiovascular disease risk (6, 7). Compared to traditional lipid parameters, non-traditional lipid parameters have demonstrated greater predictive value for cardiovascular disease risk (8–11). Based on these traditional lipid parameters, non-traditional lipid parameters can be calculated, including the Atherogenic Index of Plasma (AIP), non-high-density lipoprotein cholesterol (non-HDL-c), Lipoprotein Combine Index (LCI), Remnant Cholesterol (RC), Atherogenic Coefficient (AC), Castelli's Risk Index-I (CRI-I), and Castelli's Risk Index-II (CRI-II). These non-traditional lipid parameters expand the scope of the lipid profiles, thereby providing a more accurate reflection of the balance between atherogenic and anti-atherogenic lipoproteins in the body. Nevertheless, the relationship between non-traditional lipid parameters and moderate-to-severe ICAS and ECAS remains incompletely understood.

This study aims to analyze the differences of non-traditional lipid parameters in patients with moderate-to-severe ICAS and ECAS at high risk of stroke and to explore their relationship with stroke events. To investigate the correlation among the location (ICAS/ECAS/combined ICAS and ECAS) and distribution (anterior/posterior/combined anterior and posterior circulation) of the stenosis and the presence or absence of symptomatic events (defined as the presence or absence of stroke or transient ischemic attack [TIA] episodes within the last month), in order to facilitate a more comprehensive assessment of the risk of cardiovascular and cerebrovascular diseases and to effectively guide clinical stroke prevention and treatment strategies.

## Subjects and methods

This study utilized a single-center, retrospective clinical design. The methodology for patient selection, clinical data collection, and analysis has been detailed in an article published previously (12).

## Patient selection

This study included middle-aged and elderly patients who underwent cerebrovascular digital subtraction angiography (DSA) at the Department of Neurology in Zhongnan Hospital of Wuhan University from January 2017 to October 2021. Patients with moderate (50%−69%) and severe stenosis (70%−99% or occlusion) were selected for inclusion, while those with no stenosis, mild stenosis (<49%), or stenosis due to other causes were excluded. The exclusion criteria were as follows: non-Chinese nationality; age younger than 45-year-old; incomplete DSA data or laboratory tests; evidence of cardiogenic embolisms, such as the history of atrial fibrillation; artery stenosis caused by dissection; hemorrhagic stroke; subarachnoid hemorrhage; moyamoya disease; fibromuscular dysplasia; arteriovenous malformation; aneurysm; signs of acute infection; tumor; hematological disorders; severe liver and kidney function impairment. The study was approved by the Clinical Research Ethics Committee of Zhongnan Hospital of Wuhan University (ref. no.: 2,022,106K).

## Data collection and analysis

The hospital information management system was used to extract essential clinical data, including demographic characteristics (gender, age), past medical history (hypertension, diabetes, ischemic stroke), smoking and drinking habits, and lipid profiles (LDL-c, TC, TG, and HDL-c) within 24 hours of admission. The non-lipid parameters are calculated as follows:

AIP = lg (TG/HDL-c) (13);
non-HDL-C = TC–HDL-c (14);
AC = non-HDL-c/HDL-c (15);
CRI-I = TC/HDL-c (15);
CRI-II = LDL-c/HDL-c (15);
LCI = (TC*TG*LDL-c)/HDL-c (16);
RC = TC–HDL-c–LDL-c (17).

In this study, the DSA data of all patients were independently evaluated by two neuro-interventionalists with more than 5 years of experience in DSA image interpretation. In the event of a discrepancy, a third neuro-interventionalist was consulted to confirm the results. The intracranial arteries include the C6–C7 segments of the internal carotid artery (ICA), the M1–M2 segments of the middle cerebral artery, the A1–A2 segments of the anterior cerebral artery, the P1–P2 segments of the posterior cerebral artery, the V4 segment of the vertebral artery, and the basilar artery. The extracranial arteries included the subclavian artery, the V1–V3 segments of the vertebral artery, the common carotid artery, and the C1–C5 segments of the ICA. Patients presenting with ICAS only, ECAS only, or both were categorized into the ICAS group, ECAS group, or the combined ICAS and ECAS group. The patients were classified into three groups based on the distribution of stenosis: the anterior circulation atherosclerotic stenosis group, the posterior circulation atherosclerotic stenosis group, and the combined anterior and posterior circulation atherosclerotic stenosis group. The degree of stenosis was evaluated according to the methodology outlined in the Warfarin-Aspirin Symptomatic Intracranial Disease Study (4), where the degree of stenosis is calculated as follows: degree of stenosis (%) = (1 – diameter of the narrowest point of the narrowed segment/diameter of the proximal normal vessel) × 100%. Patients exhibiting moderate stenosis (50–69%) and severe stenosis (70–99% or occlusion) were included in the analysis. Patients were assigned to the symptomatic stenosis group if they exhibited symptoms consistent with a TIA and/or ischemic stroke in the region of the stenotic artery within the subsequent 30 days, as defined by the SAMMPRIS and VISSIT studies (18, 19). TIA and ischemic stroke were diagnosed in accordance with the criteria set forth by the American Heart Association/American Stroke Association (AHA/ASA) (20).

## Statistical analysis

The baseline characteristics of the patients, including gender, age, past medical history, and lipid levels, were compared among the groups. For Gaussian distributions, continuous variables were expressed as mean ± standard deviation. Conversely, for non-Gaussian distributions, median and interquartile range (IQR) were applied. Categorical variables were expressed as frequencies (proportions), and comparisons were made using the Kruskal–Wallis test and chi-square test. *P*-values were two-sided, and the significance level was set at *P* < 0.05. A logistic regression model was employed to examine the relationship among non-traditional lipid parameters and ICAS and ECAS with symptoms. Restricted spline-like bar graphs were employed to model the non-linear relationship between non-traditional lipid parameters and symptomatic stenosis. All statistical analyses were conducted using the statistical software R (version 4.0.5) (20).

## Results

### Baseline characteristics

**Figure 1:**
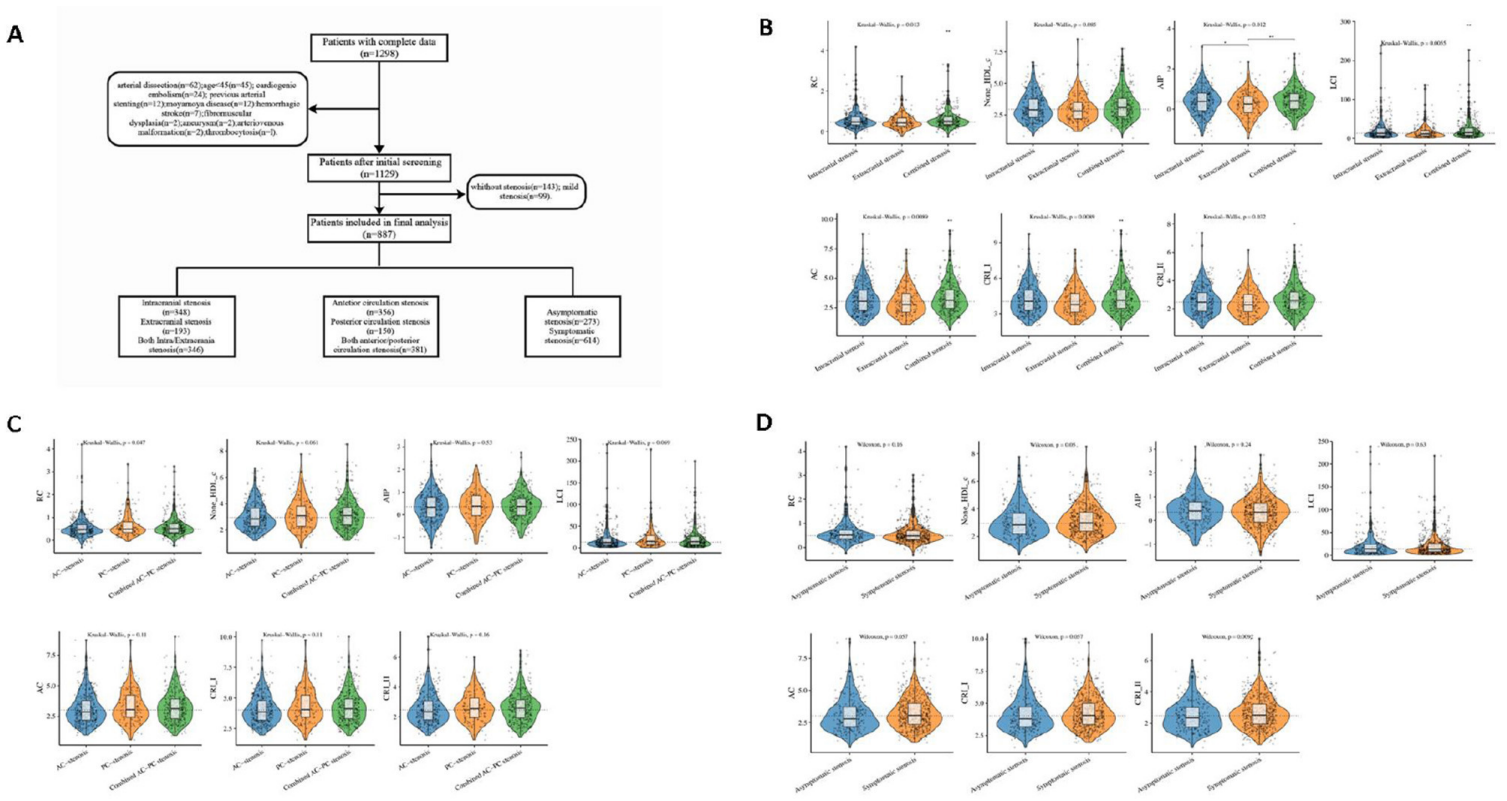
Flowchart and violin plots. **(A)** Flowchart. **(B–D)** The violin plots demonstrating the distribution of the non-traditional lipid parameters among patients in different groups. **(B)** intracranial stenosis, extracranial stenosis, and combined intracranial and extracranial stenosis. **(C)** anterior circulation (AC) stenosis, posterior circulation (PC) stenosis and combined anterior/posterior (AC-PC) stenosis. **(D)** Asymptomatic stenosis, and symptomatic stenosis.

As illustrated in the flowchart (Figure 1A), a total of 887 patients enrolled in the study. As shown in Table 1, the median age of the included patients was 62 years (IQR 56–68), with 72.7% being male. Among the non-traditional lipid parameters, the median of AIP was 0.4 (−0.1 to 0.8), non-HDL-c was 3.0 (2.3–3.7), LCI was 13.3 (7.4–25.4), RC was 0.5 (0.3–0.7), AC was 3.0 (2.3–3.9), CRI-I was 4.0 (3.3–4.9), and CRI-II was 2.5 (1.9–3.1). The total number of patients with ICAS, ECAS, and combined ICAS/ECAS was 348, 193, and 346, respectively. The number of patients with anterior circulation atherosclerotic stenosis, posterior circulation atherosclerotic stenosis, and combined anterior and posterior circulation atherosclerotic stenosis were 356, 150, and 381, respectively. Two hundred and seventy-three patients were asymptomatic, while 614 patients exhibited symptoms. The non-traditional lipid parameters among patients with different arterial stenosis status was shown by violin plots in Figure 1.

**Table 1:** Comparison of factors among patients with moderate-to-severe atherosclerotic stenosis in different locations of cerebral vessels.

	**All (*N =* 887)**	**Intracranial stenosis alone (*N =* 348)**	**Extracranial stenosis alone (*N =* 193)**	**Combined Intracranial/extracranial stenosis (*N =* 346)**	**Overall (*P*-value)**	**ICAS vs ECAS (*P*-value)**	**ICAS vs. Combined ICAS/ECAS (*P*-value)**	**ECAS vs. Combined ICAS/ECAS (*P*-value)**
Male, *N* (%)	645 (72.7%)	224 (64.4%)	160 (82.9%)	261 (75.4%)	**<0.001**	**<0.001**	**0.003**	0.057
Age, median (IQR)	62.0 [56.0–68.0]	58.0 [53.0–65.0]	65.0 [59.0–70.0]	63.0 [57.0–69.8]	**<0.001**	**<0.001**	**<0.001**	0.180
Drink, *N* (%)	120 (13.5%)	44 (12.6%)	29 (15.0%)	47 (13.6%)	0.739	0.799	0.799	0.799
smoking, *N* (%)	286 (32.2%)	99 (28.4%)	66 (34.2%)	121 (35.0%)	0.149	0.294	0.233	0.931
**Medical history**								
Hypertension, *N* (%)	635 (71.6%)	238 (68.4%)	137 (71.0%)	260 (75.1%)	0.140	0.597	0.176	0.514
Diabetes, *N* (%)	289 (32.6%)	100 (28.7%)	57 (29.5%)	132 (38.2%)	**0.018**	0.923	0.032	0.083
Pre-stroke, *N* (%)	219 (24.7%)	84 (24.1%)	49 (25.4%)	86 (24.9%)	0.945	0.973	0.973	0.973
TC, median (IQR), mmol/L	4.0 [3.3–4.8]	4.0 [3.2–4.8]	3.8 [3.3–4.5]	4.1 [3.4–4.8]	0.251	0.404	0.404	0.264
TG, median (IQR), mmol/L	1.4 [1.0–1.9]	1.4 [1.0–1.9]	1.3 [1.0–1.7]	1.4 [1.1–1.9]	**0.013**	0.085	0.258	0.008
HDL-c, median (IQR), mmol/L	1.0 [0.8–1.1]	1.0 [0.8–1.1]	1.0 [0.9–1.2]	1.0 [0.8–1.1]	0.072	0.076	0.866	0.076
LDL-c, median (IQR), mmol/L	2.4 [1.9–3.1]	2.3 [1.8–3.1]	2.4 [1.8–2.9]	2.5 [1.9–3.1]	0.298	0.666	0.405	0.405
RC, median (IQR), mmol/L	0.5 [0.3–0.7]	0.5 [0.3–0.7]	0.4 [0.3–0.7]	0.5 [0.4–0.7]	**0.013**	0.071	0.255	0.010
Non-HDL-c, median (IQR), mmol/L	3.0 [2.3–3.7]	2.9 [2.3–3.8]	2.8 [2.2–3.5]	3.1 [2.4–3.8]	0.085	0.256	0.256	0.076
AIP, median (IQR)	0.4 [−0.1–0.8]	0.4 [−0.1–0.8]	0.3 [−0.2–0.6]	0.4 [<0.1–0.8]	**0.012**	**0.042**	0.433	**0.009**
LCI, median (IQR)	13.3 [7.4-25.4]	13.0 [7.3-25.9]	11.4 [6.8-20.5]	14.8 [8.6-26.6]	**0.005**	0.089	0.121	**0.004**
AC, median (IQR)	3.0 [2.3–3.9]	3.0 [2.3–4.0]	2.8 [2.1–3.7]	3.1 [2.5–4.0]	**0.009**	0.069	0.213	**0.006**
CRI-I, median (IQR)	4.0 [3.3–4.9]	4.0 [3.3-5.0]	3.8 [3.1–4.7]	4.1 [3.5-5.0]	**0.009**	0.069	0.213	**0.006**
CRI-II, median (IQR)	2.5 [1.9–3.1]	2.5 [1.8–3.1]	2.3 [1.8–3.0]	2.6 [2.0–3.2]	**0.032**	0.184	0.184	**0.030**

TC, total cholesterol; TG, triglycerides; HDL-c, high-density lipoprotein cholesterol; LDL-c, low-density lipoprotein cholesterol; RC, residual cholesterol; Non- HDL-c, non-high-density lipoprotein cholesterol; AIP, plasma atherosclerotic index; LCI, lipoprotein binding index; AC, coefficient of atherosclerosis; CRI-I, Castelli Index-I; CRI-II, Castelli Index-II; IQR, interquartile range; Castelli index-I; CRI-II, Castelli index-II; IQR, interquartile range.

P-values are used among ICAS/ECAS/combined ICAS and ECAS. Bold indicates valid values (P < 0.05).

### The relationship of non-traditional lipid parameters and stenosis location

In the stenosis location, traditional lipid parameters (TC, TG, HDL-c, and LDL-c) revealed no significant differences between ICAS and ECAS. In contrast, among the non-traditional lipid parameters, the differences in AIP, LCI, RC, AC, CRI-I, and CRI-II were statistically significant in the three groups (*P* = 0.012, 0.005, 0.013, 0.009, 0.009, 0.032, respectively). In comparison to the other two groups, the combined ICAS/ECAS group exhibited more pronounced elevations in non-traditional lipid parameters. Moreover, non-traditional lipid parameters were generally higher in ICAS patients than in those with ECAS alone, and only AIP demonstrated a statistically significant difference. The baseline data and results are presented in Table 1.

### The relationship of non-traditional lipid parameters and stenosis distribution

Baseline clinical data and laboratory measurements in patients with different stenosis distributions are shown in Table 2. The RC differed meaningfully (*P* = 0.047) among the anterior circulation atherosclerotic stenosis group (median, 0.5; IQR, 0.3–0.7), the posterior circulation atherosclerotic stenosis group (median, 0.5; IQR, 0.3–0.8), and the combined anterior/posterior circulation atherosclerotic stenosis group (median, 0.5; IQR, 0.3–0.7).

**Table 2:** Comparison of non-traditional lipid parameters among patients with moderate-to-severe atherosclerotic stenosis in different cerebral circulations.

	**All (*N =* 887)**	**Anterior circulation stenosis (*N =* 356)**	**Posterior circulation stenosis (*N =* 150)**	**Combined anterior/posterior circulation stenosis (*N =* 381)**	**Overall (*P*-value)**	**Anterior circulation stenosis vs. Posterior circulation stenosis (*P*-value)**	**Anterior circulation stenosis vs. anterior/posterior circulation stenosis (*P*-value)**	**Posterior circulation stenosis vs. anterior/posterior circulation stenosis (*P*-value)**
Male, *N* (%)	645 (72.7%)	252 (70.8%)	106 (70.7%)	287 (75.3%)	0.317	1.000	0.481	0.481
Age, median (IQR)	62.0 [56.0–68.0]	59.0 [54.0–66.0]	60.5 [54.2–67.0]	65.0 [58.0–71.0]	**<0.001**	0.335	**<0.001**	**<0.001**
Drink, *N* (%)	120 (13.5%)	49 (13.8%)	19 (12.7%)	52 (13.6%)	0.943	1.000	1.000	1.000
smoking, *N* (%)	286 (32.2%)	115 (32.3%)	39 (26.0%)	132 (34.6%)	0.159	0.290	0.552	0.208
**Medical history**								
Hypertension, *N* (%)	635 (71.6%)	217 (61.0%)	108 (72.0%)	310 (81.4%)	**<0.001**	**0.024**	**<0.001**	**0.024**
Diabetes, *N* (%)	289 (32.6%)	97 (27.2%)	50 (33.3%)	142 (37.3%)	**0.015**	0.306	**0.014**	0.453
Pre-stroke, *N* (%)	219 (24.7%)	85 (23.9%)	35 (23.3%)	99 (26.0%)	0.734	0.987	0.902	0.902
TC, median (IQR), mmol/L	4.0 [3.3–4.8]	3.8 [3.2–4.7]	4.0 [3.4-5.0]	4.1 [3.4–4.7]	0.072	0.090	0.090	0.523
TG, median (IQR), mmol/L	1.4 [1.0–1.9]	1.4 [1.0–1.9]	1.5 [1.1–2.0]	1.4 [1.0–1.9]	0.252	0.356	0.369	0.369
HDL-c, median (IQR), mmol/L	1.0 [0.8–1.1]	1.0 [0.8–1.2]	1.0 [0.8–1.2]	1.0 [0.9–1.1]	0.975	0.939	0.939	0.939
LDL-c, median (IQR), mmol/L	2.4 [1.9–3.1]	2.3 [1.8–3.0]	2.5 [1.9–3.2]	2.5 [1.9–3.0]	0.212	0.277	0.277	0.846
RC, median (IQR), mmol/L	0.5 [0.3–0.7]	0.5 [0.3–0.7]	0.5 [0.3–0.8]	0.5 [0.3–0.7]	**0.047**	0.073	0.073	0.693
Non-HDL-c, median (IQR), mmol/L	3.0 [2.3–3.7]	2.8 [2.3–3.7]	3.1 [2.3–3.8]	3.1 [2.4–3.7]	0.061	0.081	0.081	0.674
AIP, median (IQR)	0.4 [−0.1–0.8]	0.3 [−0.1–0.8]	0.4 [<0.1–0.8]	0.4 [<0.1–0.7]	0.532	0.530	0.530	0.530
LCI, median (IQR)	13.3 [7.4-25.4]	12.4 [7.3-22.2]	15.0 [7.7-28.5]	14.4 [7.5-26.2]	0.069	0.100	0.100	0.621
AC, median (IQR)	3.0 [2.3–3.9]	2.9 [2.2–3.8]	3.1 [2.4–4.2]	3.1 [2.3–3.9]	0.111	0.129	0.129	0.734
CRI-I, median (IQR)	4.0 [3.3–4.9]	3.9 [3.2–4.8]	4.1 [3.4-5.2]	4.1 [3.3–4.9]	0.111	0.129	0.129	0.734
CRI-II, median (IQR)	2.5 [1.9–3.1]	2.4 [1.8–3.1]	2.6 [2.0–3.3]	2.6 [1.9–3.2]	0.159	0.296	0.213	0.880

IQR, interquartile range.

P-value for comparison among anterior circulation atherosclerotic stenosis/posterior circulation atherosclerotic stenosis/combined anterior and posterior circulation atherosclerotic stenosis. Bold indicates valid values (P < 0.05).

### The relationship of non-traditional lipid parameters and asymptomatic or symptomatic stenosis

In this study, if patients exhibited symptoms consistent with a stroke or TIA caused by an ischemic lesion in a relevant artery with ≥50% stenosis within the subsequent 30 days, they were assigned into the symptomatic stenosis group. As demonstrated in Table 3, patients with moderate-to-severe atherosclerotic stenosis who exhibited symptoms were older and had a higher proportion of males, smoking, drinking, and hypertension compared to those without symptoms. In terms of lipid metabolism, a logistic regression analysis revealed that CRI-II (odds ratio [OR] = 1.20, confidence interval [CI] = 1.03–1.40, *P* = 0.009) and LDL-c (OR = 1.21, CI = 1.03–1.42, *P* = 0.011) were elevated in patients with symptomatic stenosis compared to those with asymptomatic stenosis. The observed discrepancies were statistically significant.

**Table 3:** Comparison of non-traditional lipid parameters among patients with and without symptomatic stenosis.

	**All (*N =* 887)**	**Asymptomatic stenosis (*N =* 273)**	**Symptomatic stenosis (*N =* 614)**	**Overall (*P*-value)**	**OR**	***P*-ratio**
Male, *N* (%)	645 (72.7%)	183 (67.0%)	462 (75.2%)	**0.014**	1.49 [1.09–2.04]	0.012
Age, median (IQR)	62.0 [56.0–68.0]	64.0 [57.0–71.0]	61.0 [55.0–67.0]	**<0.001**	0.97 [0.95–0.98]	<0.001
Drink, *N* (%)	120 (13.5%)	23 (8.4%)	97 (15.8%)	**0.004**	2.03 [1.28–3.35]	0.002
smoking, *N* (%)	286 (32.2%)	67 (24.5%)	219 (35.7%)	**0.001**	1.70 [1.24–2.36]	0.001
**Medical history**						
Hypertension, *N* (%)	635 (71.6%)	211 (77.3%)	424 (69.1%)	**0.015**	0.66 [0.47–0.91]	0.011
Diabetes, *N* (%)	289 (32.6%)	91 (33.3%)	198 (32.2%)	0.810	0.95 [0.70–1.29]	0.749
Pre-stroke, *N* (%)	219 (24.7%)	70 (25.6%)	149 (24.3%)	0.724	0.93 [0.67–1.29]	0.659
TC, median (IQR), mmol/L	4.0 [3.3–4.8]	3.8 [3.2–4.7]	4.0 [3.3–4.8]	0.071	1.09 [0.96–1.24]	0.191
TG, median (IQR), mmol/L	1.4 [1.0–1.9]	1.5 [1.0–2.0]	1.4 [1.0–1.9]	0.060	0.88 [0.77–1.00]	0.048
HDL-c, median (IQR), mmol/L	1.0 [0.8–1.1]	1.0 [0.8–1.1]	1.0 [0.8–1.2]	0.924	1.09 [0.61–1.96]	0.765
LDL-c, median (IQR), mmol/L	2.4 [1.9–3.1]	2.3 [1.7–2.9]	2.4 [1.9–3.1]	**0.011**	1.21 [1.03–1.42]	0.020
RC, median (IQR), mmol/L	0.5 [0.3–0.7]	0.5 [0.3–0.7]	0.5 [0.3–0.7]	0.157	0.76 [0.55–1.05]	0.096
Non-HDL-c, median (IQR), mmol/L	3.0 [2.3–3.7]	2.8 [2.2–3.7]	3.0 [2.4–3.8]	0.050	1.09 [0.96–1.25]	0.191
AIP, median (IQR)	0.4 [−0.1–0.8]	0.4 [<0.1–0.8]	0.3 [−0.1–0.7]	0.238	0.83 [0.66–1.05]	0.115
LCI, median (IQR)	13.3 [7.4-25.4]	13.9 [6.5-24.4]	13.2 [7.7-25.8]	0.629	1.00 [0.99–1.00]	0.306
AC, median (IQR)	3.0 [2.3–3.9]	2.8 [2.2–3.7]	3.1 [2.4–4.0]	0.057	1.08 [0.96–1.21]	0.215
CRI-I, median (IQR)	4.0 [3.3–4.9]	3.8 [3.2–4.7]	4.1 [3.4-5.0]	0.057	1.08 [0.96–1.21]	0.215
CRI-II, median (IQR)	2.5 [1.9–3.1]	2.3 [1.8–3.0]	2.5 [2.0–3.2]	**0.009**	1.20 [1.03–1.40]	**0.018**

IQR, interquartile range.

P-value for comparison between asymptomatic and symptomatic stenosis. Bold indicates valid values (P < 0.05).

After adjusting for confounding variables, including age, sex, smoking, drinking, medical history of hypertension, diabetes, and stroke, as well as the location and distribution of stenosis, CRI-II remained an independent risk factor for symptomatic stenosis (OR = 1.18, CI 1.00–1.38, *P* = 0.047). The results of this study are presented in Table 4. Multivariate spline regression modeling, as shown in Figure 2, demonstrated that an elevated CRI-II was associated with an increased risk of stroke events in patients with moderate-to-severe atherosclerotic stenosis and this correlation is not non-linear (*P* = 0.5020).

**Table 4:** The relationship of CRI-II index and stroke events in patients with moderate-to-severe stenosis.

	Model 1^*^			**Model 2^†^**			**Model 3^#^**		
	**OR**	**(95% CI)**	***P*-value**	**OR**	**(95% CI)**	***P*-value**	**OR**	**(95% CI)**	***P*-value**
**Asymptomatic stenosis (*****N** =* **273)**									
**CRI-II**	Reference	–	–	Reference	–	–	Reference	–	–
**Symptomatic stenosis (*****n*** = **614)**									
**CRI-II**	1.20	[1.03–1.40]	**0.018**	1.15	[1.01–1.34]	**0.042**	1.18	[1.00–1.38]	**0.047**

CRI-II index, Castelli Index-II, the ratio of LDL-c to HDL-c.

* Model 1: unadjusted.

† Model 2: adjusted for age and sex.

# Model 3: adjusted for age, sex, smoking, drinking, medical history of hypertension, diabetes and stroke, stenosis location, and stenosis distribution.

**Figure 2:**
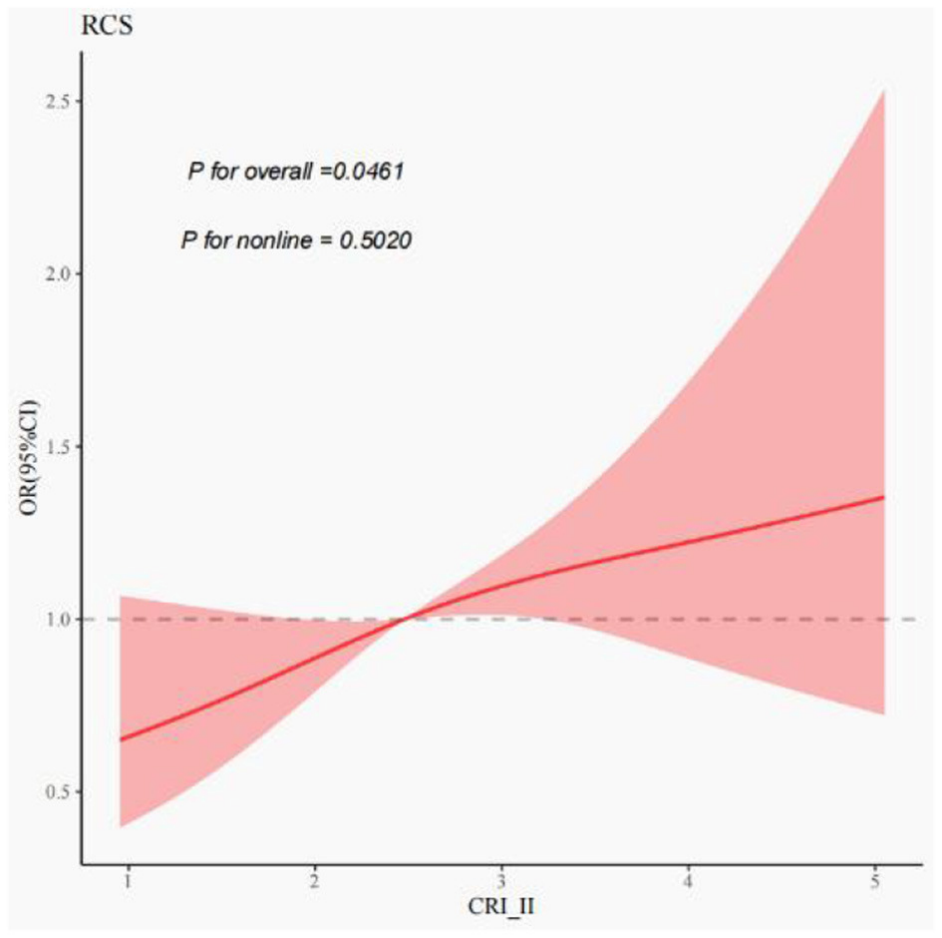
Correlation of CRI-II with stroke events in moderate-to-severe stenosis. Restricted cubic spines of the relationship between non-traditional lipid parameters and stroke events. The model adjusted for age, sex, smoking, drinking, medical history of hypertension, diabetes and stroke, stenosis location, and stenosis distribution.

### Comparison of non-traditional lipid parameters in a non-stenotic group vs. patients with moderate-to-severe stenosis

In order to generalize the findings to a more extensive population and to provide a more comprehensive illustration of the role of non-traditional lipid parameters as indicators of risk for cerebrovascular stenosis and stroke, a comparative analysis was conducted between non-traditional lipid parameters in a non-stenotic group and moderate-to-severe stenosis patients. This analysis revealed that HDL-c, AIP, AC, CRI-I, and CRI-II remained statistically significant (*P* = 0.001, 0.039, 0.042, 0.042, 0.040, respectively) (Supplementary Table S1).

## Discussion

Previous studies have demonstrated a strong correlation between the degree of stenosis and the risk of stroke. Specifically, researches indicate that in carotid stenosis, the risk of stroke is highly dependent on the degree of stenosis, with the incidence of ipsilateral stroke progressively increasing as stenosis worsens (21–23). This study is a retrospective analysis of patients with moderate-to-severe stenosis, a population at higher risk for stroke. By analyzing clinical data and cerebrovascular DSA examinations, the correlation between non-traditional lipid parameters and moderate-to-severe ICAS and ECAS was evaluated. The results suggest that: (1) Lipid parameters for ICAS are generally higher than those for ECAS. AIP, LCI, RC, AC, CRI-I, and CRI-II are more strongly correlated with extensive cerebrovascular lesions; (2) RC shows statistically significant differences among groups in the anterior, posterior, and combined anterior and posterior circulation atherosclerotic stenosis; (3) CRI-II was identified as an independent risk factor for symptomatic atherosclerotic stenosis, with elevated CRI-II levels linked to an increased risk of stroke events in patients with moderate-to-severe atherosclerotic stenosis.

A single traditional lipid parameter is insufficient to provide a comprehensive understanding of lipid component metabolism interactions. Non-traditional lipid parameters (AIP, non-HDL-c, RC, AC, LCI, CRI-I, CRI-II), calculated from traditional lipid parameters, have emerged as potential alternative predictors of cardiovascular risk. These non-traditional lipid parameters have been shown to be independent risk factors for carotid plaque susceptibility in patients with acute ischemic stroke, with a positive correlation observed between these parameters and the degree of carotid plaque stenosis (24). Furthermore, non-traditional lipid parameters demonstrated superior efficacy in assessing the risk of bleeding after endovascular treatment in acute ischemic stroke compared to traditional lipid parameters (25). However, the relationship between non-traditional lipid parameters and ICAS and ECAS, particularly in terms of location, distribution, and the presence or absence of symptoms, remains unclear.

In this study, we compared the non-traditional lipid profiles of patients with moderate-to-severe stenosis in different locations and finally found that found statistically significant differences in AIP, LCI, RC, AC, CRI-I, and CRI-II. And the elevation of non-traditional lipid parameters was most pronounced when combined ICAS and ECAS were present. Consequently, non-traditional lipid parameters (AIP, LCI, RC, AC, CRI-I, and CRI-II) offer optimal utility in identifying extensive stenosis of the cerebral vasculature. This may be attributable to patients with both ICAS and ECAS presenting with a heavier lipid burden, more severe metabolic dysfunction and chronic inflammation, and more pronounced hemodynamic superimposed effects of multivessel stenosis (26). Compared with patients with ECAS alone, the non-traditional lipid parameters were observably elevated in patients with ICAS alone (except for LDL-c), especially AIP. AIP is defined as the logarithmic ratio of TG to HDL-c, reflecting the balance between the actual concentrations of plasma TG and HDL-c (9, 13). Our data indicate that AIP and TG are elevated in ICAS compared to ECAS, suggesting that elevated AIP and TG may have a greater impact on the intracranial vessels. This finding aligns with previous studies that lipid disorders are more strongly correlated with the severity of intracranial stenosis (27). It can be posited that AIP may act as an independent risk factor for the development of ICAS and ECAS.

This study also examined the relationship between the distribution of stenosis and non-traditional lipid parameters. The results demonstrated statistically significant differences among the three groups for RC only. Nevertheless, no discrepancy was observed when the anterior and posterior circulation groups were evaluated separately. Consequently, the clinical significance of non-traditional lipid parameters in stenosis distribution is relatively minor. This may be attributed to the limited amount of available clinical data. Furthermore, the impact of vascular anatomy, lipid sensitivity, collateral circulation, and hemodynamic factors must be given due consideration (28, 29).

As previously mentioned, the study found that CRI-II is a more valuable predictor of extensive lesions of atherosclerosis (combined ICAS/ECAS). Shun et al. have also supported that CRI-II is more valuable in predicting ICAS or combined ICAS/ECAS lesions (9). The possible reason for this result is that CRI-II is a ratio between LDL-c (major atherogenic lipoprotein) and HDL-c (anti-atherogenic lipoprotein), reflecting systemic lipid metabolism imbalances rather than local vascular specificity and revealing more sensitive to extensive atherosclerosis (e.g., combined ICAS/ECAS).

Equally important, this study demonstrated that CRI-II levels were elevated in patients with symptomatic stenosis in compared to those with asymptomatic stenosis. After adjusting for various confounding variables, including age, sex, smoking, and alcohol consumption, as well as hypertension, diabetes mellitus, history of stroke, location of stenosis, and distribution of stenosis, the CRI-II remained an independent risk factor for symptomatic stenosis. Subsequent multivariate spline regression models showed that the CRI-II was associated with the risk of ischemic stroke due to moderate-to-severe stenosis and this correlation is not non-linear. That is, as CRI-II elevated, the risk of stroke events increased progressively. Besides, a large prospective cohort study by Zhang et al. found CRI-II to be a superior predictor of stroke status in hypertensive patients (30). A recent meta-analysis also suggested that higher CRI-II levels were significantly associated with an increased risk of ischemic stroke (31). Therefore, CRI-II may serve as a more refined predictor of ischemic stroke.

Furthermore, CRI-II is anticipated to emerge as a standard for the evaluation of treatment and prognosis in cases of ischemic stroke. Ryu et al. demonstrated that enhancement of CRI-II is correlated with a diminished risk of in-stent restenosis (32), indicating that CRI-II may play an important role in the prevention of in-stent restenosis in subjects undergoing intracranial stent placement. As previously indicated, the CRI-II represents the proportion of LDL-c in relation to HDL-c (33–35). The Atherosclerosis Risk In Communities (ARIC) study demonstrated that increased LDL-c levels and decreased HDL-c levels were associated with the prevalence of symptomatic intracranial atherosclerotic stenosis (36). In our study, LDL-c levels were also markedly enhanced in patients with symptomatic stenosis. Up to the present moment, the reduction of LDL-c levels has been a key focus in routine clinical treatment (37). A reduction in LDL-c levels by 30 mg/dL has been verified to result in a 40–50% reduction in the incidence of major ischemic events (38, 39). For individuals with more than 50% carotid plaque stenosis and no history of ischemic stroke, the use of statins is advised to maintain LDL-c levels below 1.8 mmol/L, regardless of dyslipidemia or not (40, 41). Non-traditional lipid parameters, particularly CRI-II, offer an effective means of monitoring the efficacy of the therapy of LDL-c lowering. On the other hand, a multicenter study by Kim et al. provided new insights into the value of CRI-II. They analyzed non-traditional lipid profiles in ischemic stroke patients on statin therapy with admission LDL-c < 100 mg/dl found that CRI-II performed best and that there was a linear relationship between CRI-II and increased risk of vascular events in 1 year (42). This suggests that the CRI-II may be useful in predicting the risk of subsequent vascular events in patients who have had an ischemic stroke, despite good control of LDL with statin therapy.

Interestingly, smoking and drinking have been identified as an important contributing factor to the development of stroke (43, 44). In this study, the proportion of smoking and alcohol consumption was observed to be relatively lower in ICAS patients compared to ECAS patients. This finding may suggest that the development of ICAS is more dependent on lipid metabolism disorders and inflammation than on behavioral factors alone (26, 27). Moreover, a significantly higher prevalence of diabetes and hypertension in the patients with moderate-to-severe stenosis compared to non-stenotic group. The synergistic effect of diabetes and hypertension has been demonstrated to result in an increased cardiovascular risk, and is thus considered a significant risk factor for the progression of atherosclerotic stenosis (45–47). Our study re-emphasizes the importance of integrated management of blood glucose, blood pressure and lipids in the clinical management of cardiovascular disease. We also compared non-traditional lipid parameters in non-stenosis and moderate-to-severe stenosis patients and finally found that AIP, AC, CRI-I, and CRI-II remained statistically significant. This finding may indicate that an imbalance in lipid metabolism plays a pivotal role in the progression of atherosclerosis and that non-traditional lipid parameters are potential to serve as effective predictors of atherosclerotic stenosis progression and the risk of stroke occurrence.

Several limitations merit consideration in our study. Firstly, the research was conducted at a single center and involved patients from a Chinese hospital, potentially introducing selection bias. Consequently, further validation in larger external populations is warranted. Secondly, the study population was predominantly Han Chinese. Given that lipid parameters were associated with ethnicity, dietary patterns, and lifestyle factors, replication studies in multiethnic cohorts are imperative. Thirdly, almost all participants received lipid-lowering agents, with interindividual variability in drug types (statins or ezetimibe), dosages, and treatment durations. This heterogeneity may affect the lipid parameters. Collectively, there is a need for further research in different ethnic groups and regions to confirm the applicability of the results to a wider population.

## Conclusion

In summary, non-traditional lipid parameters demonstrated higher predictive value in the assessment of extensive lesions of atherosclerotic stenosis, as well as the presence or absence of symptoms. CRI-II has been identified as an independent risk factor for symptomatic stenosis. These non-traditional parameters provide greater insight into ICAS and ECAS, as well as enable quantitative risk assessment. Incorporating these metrics into routine clinical practice may facilitate effective patient treatment and lifestyle modifications.

## Data Availability

The data that support the findings of this study are available from the corresponding author upon reasonable request.
