# Supplementary material for: Non-traditional lipid parameters are independent predictors of the location, distribution, and stroke events of moderate-to-severe intracranial and extracranial atherosclerotic stenosis
Source: Front Neurol. 2025 Jul 9;16:1564966. doi: 10.3389/fneur.2025.1564966 (PMC12283305; doi:10.3389/fneur.2025.1564966)
Supplement: Supplementary file 1 [file Table_1.docx]

**Supplement**

**Supplement Table1. Comparison of factors among patients with moderate-to-severe atherosclerotic stenosis in different locations of cerebral vessels.**

|  | **All(N=1030)** | **Without stenosis (N=143)** | **Moderate-to-severe stenosis( N=887)** | **Overall *P*.value** |
| --- | --- | --- | --- | --- |
| Male, N（%） | 737 (71.6%) | 92 (64.3%) | 645 (72.7%) | 0.050 |
| Age, median(IQR) | 61.5  [55.0;68.0] | 59.0  [54.0;64.5] | 62.0  [56.0;68.0] | **0.001** |
| Drink, N (%) | 142 (13.8%) | 22 (15.4%) | 120 (13.5%) | 0.641 |
| smoking, N(%) | 331 (32.1%) | 45 (31.5%) | 286 (32.2%) | 0.930 |
| **Medical history** |  |  |  |  |
| Hypertension, N(%) | 712(69.1%) | 77 (53.8%) | 635 (71.6%) | **<0.001** |
| Diabetes, N(%) | 308(29.9%) | 19 (13.3%) | 289 (32.6%) | **<0.001** |
| Pre-stroke, N(%) | 245(23.8%) | 26 (18.2%) | 219 (24.7%) | 0.112 |
| TC, median(IQR), mmol/L | 4.0 [3.3;4.8] | 4.1 [3.5;4.9] | 4.0 [3.3;4.8] | 0.198 |
| TG, median(IQR), mmol/L | 1.4 [1.0;1.9] | 1.4 [0.9;1.9] | 1.4 [1.0;1.9] | 0.688 |
| HDL-c, median(IQR), mmol/L | 1.0 [0.8;1.2] | 1.0 [0.9;1.2] | 1.0 [0.8;1.1] | **0.001** |
| LDL-c, median(IQR), mmol/L | 2.4 [1.9;3.1] | 2.5 [1.9;3.0] | 2.4 [1.9;3.1] | 0.464 |
| RC, median(IQR), mmol/L | 0.5 [0.3;0.7] | 0.5 [0.3;0.7] | 0.5 [0.3;0.7] | 0.668 |
| Non-HDL-c, median(IQR), mmol/L | 3.0 [2.3;3.7] | 3.0 [2.4;3.7] | 3.0 [2.3;3.7] | 0.596 |
| AIP, median(IQR) | 0.3 [-0.1;0.8] | 0.2 [-0.2;0.7] | 0.4 [-0.1;0.8] | **0.039** |
| LCI, median(IQR) | 13.2[7.5;24.8] | 12.0 [7.6;22.5] | 13.3 [7.4;25.4] | 0.487 |
| AC, median(IQR) | 3.0 [2.3;3.9] | 2.8 [2.2;3.6] | 3.0 [2.3;3.9] | **0.042** |
| CRI-I, median(IQR) | 4.0 [3.3-4.9] | 3.8 [3.2;4.6] | 4.0 [3.3;4.9] | **0.042** |
| CRI-II, median(IQR) | 2.5[1.9;3.1] | 2.3 [1.9;2.9] | 2.5 [1.9;3.1] | **0.040** |
